# Mcm10 proteolysis initiates before the onset of M-phase

**DOI:** 10.1186/1471-2121-11-84

**Published:** 2010-10-28

**Authors:** Manpreet Kaur, Aparna Sharma, Muntaz Khan, Ananya Kar, Sandeep Saxena

**Affiliations:** 1 National Institute of Immunology, Aruna Asaf Ali Marg, New Delhi-110067, India; 2 Department of Biochemistry, University of Delhi South Campus, Benito Juarez Road, New Delhi,-110021, India

## Abstract

**Background:**

Mcm10 protein is essential for initiation and elongation phases of replication. Human cells proteolyze Mcm10 during mitosis, presumably to ensure a single round of replication. It has been proposed that anaphase promoting complex ubiquitinates Mcm10 in late M and early G_1 _phases.

**Results:**

In contrast to the previous work, we report that the degradation of Mcm10 is initiated at the onset of mitosis. Immunoblotting and immunofluorescence assays display that Mcm10 levels are low in all phases of mitosis. We report that Mcm10 degradation is not dependent on anaphase promoting complex. Further, the proteolysis in M-phase can be independently mediated by non-overlapping regions of Mcm10, apparently employing a redundant mechanism to ensure downregulation.

**Conclusions:**

It is believed that the proteolysis of Mcm10 during mitosis is a vital mechanism to prevent aberrant initiation of replication and the present study describes the regulation of Mcm10 during this phase of the cell-cycle.

## Background

DNA replication in eukaryotes begins with the assembly of the pre-replicative complex comprising of replication initiators, the origin recognition complex, Cdc6, Cdt1, and the replicative helicase, Mcm2-7 complex [1]. Increase in the activity of cyclin dependent kinases and loading of replication factor, Mcm10 marks the transition from G_1 _to S phase. These events promote the loading of Cdc45, RPA and DNA polymerases at the replication origins to initiate DNA synthesis. The essential requirement of Mcm10 in DNA replication initiation and elongation has been exhibited across species [2-7]. Mutations in Mcm10 cause a decrease in initiation of replication, slow progression of DNA synthesis and stalling of replication forks during elongation [8]. Since Mcm10 is essential for replication initiation and elongation, its activity is regulated in a cell-cycle manner to ensure a single round of replication. *S. cerevisiae *Mcm10 protein is present in all phases of the cell cycle though its association with chromatin is regulated to ensure replication licensing [3].

Human Mcm10 protein is known to decrease in the early G_1 _phase [9]. The levels of *MCM10 *mRNA have been evaluated as the cells pass from M-phase into G_1 _phase. Though the Mcm10 protein decreased, the levels of *MCM10 *mRNA increased within the same time period. These results demonstrate that the decrease in the Mcm10 activity during the G_1 _phase is not due to decrease in transcription but because of protein turnover. In this communication, we report that proteolysis of human Mcm10 protein initiates before the onset of mitosis. In an immunoblot with anti-Mcm10 antibody, we observed that the M-phase blocked cells have downregulated the Mcm10 protein. On the basis of single cell immunofluorescence, we show that asynchronously growing cells in different phases of mitosis have reduced levels of Mcm10. Also, Mcm10 downregulation in M-phase is independent of the APC recognition motifs: the destruction and the KEN box. We demonstrate that Mcm10 degradation is not dependent on anaphase promoting complex or on its recognition motifs but rather mediated by non-overlapping regions, apparently employing a redundant mechanism to ensure downregulation.

## Results

### Cell-cycle proteolysis of Mcm10 initiates before the onset of mitosis

**Figure 1:**
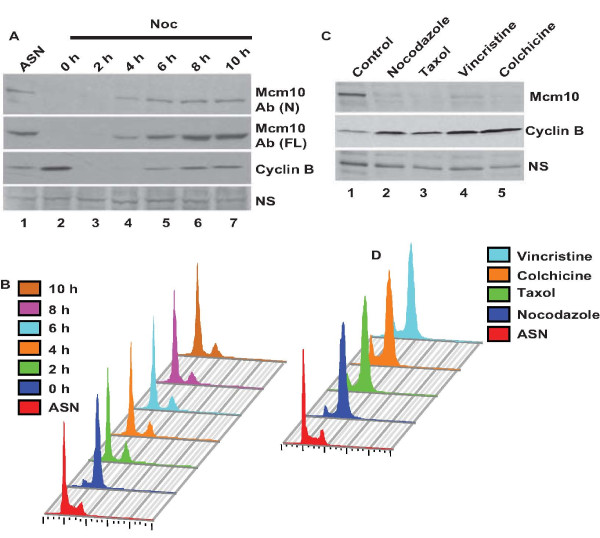
**Endogenous Mcm10 levels are low during mitosis**. U2OS cells were blocked at the mitotic phase with nocodazole for 16 h and were either harvested immediately (0 h) or released in nocodazole-free medium and then harvested at the indicated time-points and used for immunoblotting and flow cytometry. (A) Levels of endogenous Mcm10 were evaluated by Ab (N) and Ab (FL) antibody. ASN refers to samples from asynchronously growing cells while NS points to a non-specific band that displays equal protein loading in different lanes. (B) Flow cytometry of propidium iodide-stained DNA of U2OS cells, as described in (A), show an M phase block and release. The colored key denotes cells obtained at different time-points. (C) and (D) U2OS cells were blocked in mitosis with 0.5 μg/ml colchicine, 0.10 μg/ml nocodazole, 0.3 μg/ml taxol or 0.2 μg/ml vincristine for 16 h and analyzed for levels of Mcm10 (C) and DNA content (D) by immunoblotting and flow cytometry respectively. The observed mobility of endogenous Mcm10 and cyclin B was 110 kDa and 55 kDa respectively.

Published reports indicate that the nocodazole blocked cells retain Mcm10 protein which decreases in late M-phase and starts increasing 6 h after release [9]. When MG132 was added to nocodazole released cells, there was an increase in Mcm10 levels suggesting that the degradation that was occurring during the late M-phase was blocked by proteasome inhibitors. In another report by the same group, a stable HeLa cell line which expressed green fluorescent protein-tagged Mcm10 from a human cytomegalovirus immediate early promoter was established and authors observed that the GFP-Mcm10 protein was detectable by immunofluorescence during mitosis [10]. We looked at the levels of Mcm10 and observed that they were significantly reduced in nocodazole blocked U2OS cells (Figure 1A). Mcm10 appeared after 4-6 hours of release from nocodazole block. This is in contrast to the results obtained by Hanaoka and coworkers, who observed Mcm10 band by immunoblotting with anti-rabbit antibody, which was raised against 127-512 aa region of Mcm10. Therefore, we wanted to rule out that the absence of Mcm10 signal was due to limited immunoreactivity of the antibody used by us. We have used a polyclonal rabbit antibody, Ab (N), which is raised against the full-length protein, but showed weak immunoreactivity against the C-terminal protein [11]. However, similar results were obtained with another polyclonal rabbit antibody, Ab (FL), which shows immunoreactivity against all regions of Mcm10 (Figure 1A). As reported previously, the specificity of both these antibodies in immunoblotting assays have been established by RNAi (Additional file 1 and file 2: Figure S1A and S5A) [11]. In order to rule out that the decrease of Mcm10 is an artifact generated by nocodazole toxicity, we blocked the cells with other reagents in M-phase and assayed the stability of Mcm10. Vincristine binds to tubulin dimers and inhibits the assembly of microtubule structures, blocking cells in metaphase. Colchicine inhibits microtubule polymerization by binding to tubulin and thereby blocks cells in metaphase. Taxol hyper-stabilizes the beta subunit of tubulin, which is then unable to disassemble inducing a cell-cycle block at the metaphase/anaphase transition. U2OS cells blocked with any of these drugs displayed low levels of Mcm10 (Figure 1C). On the basis of the above data, we conclude that human cells have low levels of Mcm10 in mitosis.

**Figure 2:**
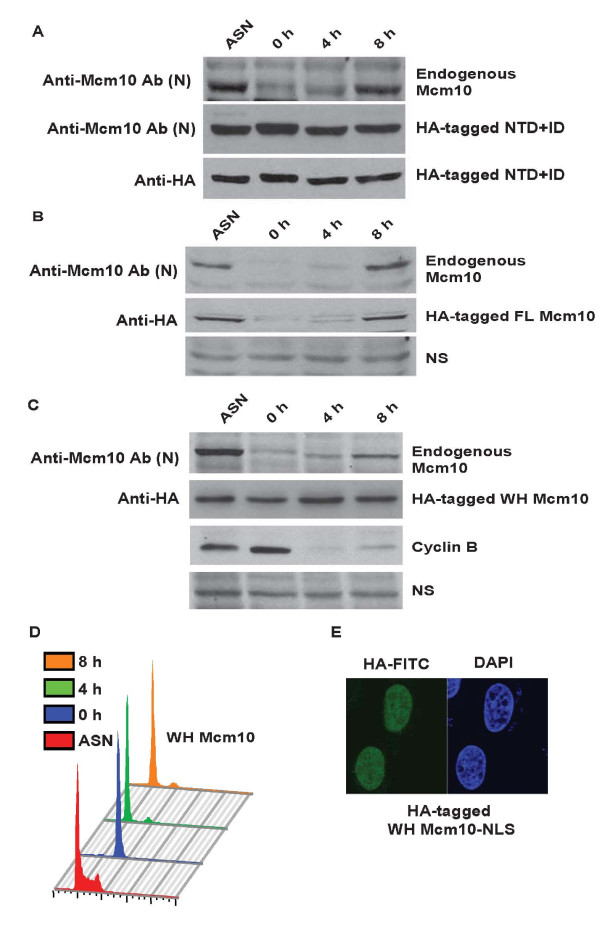
**Cell cycle-regulated degradation of Mcm10 is not dependent on APC recognition motifs: KEN and destruction box**. (A) Anti-Mcm10 antibody recognizes the mitotic forms of Mcm10. U2OS cells expressing the NTD+ID domain of Mcm10 (fused to GFP and HA epitope tags) were arrested in M-phase using nocodazole (0 h) and then harvested at the indicated time-points after release into drug-free medium. The levels of the NTD+ID domain of Mcm10 were independently evaluated by anti-HA (bottom panel) and anti-Mcm10 (middle panel) antibody. The same samples were probed with anti-Mcm10 antibody (Ab [N]) to display the levels of endogenous Mcm10 (top panel). ASN refers to samples from asynchronously growing cells. The antibody used for immunoblotting and the protein detected by it have been listed on the left and right sides of a particular western blot. The observed mobility of endogenous Mcm10 and NTD+ID domain (fused to GFP and HA epitope tags) was 110 kDa and 97 kDa respectively. (B) U2OS cells expressing HA-tagged full length Mcm10 were arrested in M-phase using nocodazole (0 h) and then harvested at the indicated time-points after release into drug-free medium. The levels of endogenous Mcm10 and HA-tagged full length Mcm10 were evaluated at different time points by anti-Mcm10 (top panel) and anti-HA (middle panel) antibody. NS points to a non-specific band that displays equal protein loading. The observed mobility of HA-tagged FL Mcm10 was approximately 115 kDa. (C) U2OS cells expressing wing-helix (WH) fragment of Mcm10 were arrested in M-phase using nocodazole (0 h) and then harvested at the indicated time-points after release into drug-free medium. The levels of endogenous Mcm10, WH fragment and cyclin B were evaluated at different time points after release from nocodazole. The observed mobility of WH fragment of Mcm10 (fused to GFP and HA epitope tags) was approximately 50 kDa. (D) The flow cytometry of propidium iodide-stained DNA of stable cell-line expressing WH fragment has been shown. The colored key denotes cells obtained at different time-points. (E) Immunofluorescence with HA antibody displays that the WH fragment of Mcm10 tagged with a nuclear localization signal is directed to the nucleus.

As reported previously, we observed that the drop in Mcm10 levels during mitosis is dependent on the proteasome (Additional file 3: Figure S2B). To rule out that loss of recognition of Mcm10 during mitosis could be due to epitope masking, we established that the Mcm10 antibody recognizes the mitotic forms of Mcm10. Since Mcm10 is naturally proteolyzed during mitosis we expressed the HA-tagged NTD+ID domain which is resistant to cell cycle-regulated degradation and tested whether the anti-Mcm10 antibody (Ab [FL]) recognizes this forms of Mcm10 during M-phase. U2OS cells expressing the NTD+ID domain of Mcm10 were blocked with nocodazole, released and harvested at regular intervals in order to collect cells in different phases of the cell cycle (Figure 2A). As observed previously, an anti-HA antibody immunoblot confirmed that the NTD+ID domain of Mcm10 was resistant to degradation in nocodazole blocked cells. An anti-Mcm10 antibody immunoblot displayed that the levels of endogenous Mcm10 levels were low in M phase and increased around 8 h after nocodazole release but the NTD+ID domain recognized through the same antibody did not show a decrease in levels, displaying that the Mcm10 antibody recognizes the mitotic forms of Mcm10.

### Single-cell imaging displays reduced levels of Mcm10 during mitosis

**Figure 3:**
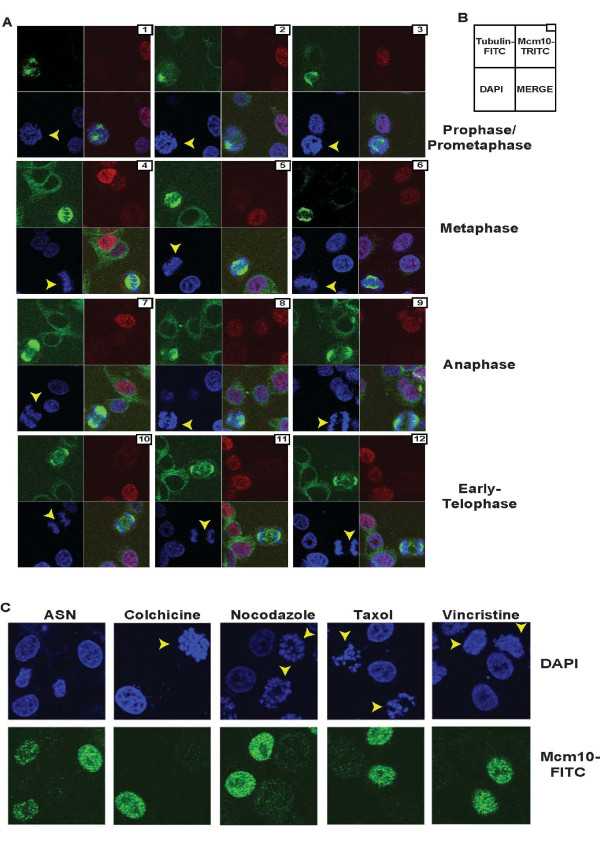
**All phases of mitosis show low levels of endogenous Mcm10**. (A) and (B) For evaluating subcellular localization of endogenous proteins, HeLa cells were fixed in formaldehyde, permeabilized with Triton X-100 and DNA was stained with 4, 6-diamidino-2-phenylindole (DAPI). To visualize Mcm10, immunofluorescence was performed using a rabbit polyclonal anti-Mcm10 antibody in combination with an anti-rabbit TRITC secondary antibody while microtubules were visualized with a mouse monoclonal antibody to alpha-tubulin conjugated to FITC. From unperturbed asynchronously growing culture, HeLa cells that are in prophase, metaphase, anaphase and telophase were identified and have been marked by arrowheads in panel (A). The arrangement of immunofluorescence images obtained from a single field has been illustrated in panel (B) and the bottom right square of each field is a merge of DAPI, FITC and TRITC images. Field numbers have been marked at the top-right corner of each field. (C) To obtain cells arrested in prometaphase (shown by arrowheads), HeLa cells were treated with colchicine, nocodazole, taxol or vincristine as described in Figure 1 and evaluated for Mcm10 levels by immunofluorescence.

We also looked at Mcm10 levels in single cells as they passed through interphase and various phases of mitosis. HeLa cells were fixed and visualized for endogenous Mcm10 using rabbit polyclonal anti-Mcm10 antibody, Ab (N), in combination with an anti-rabbit TRITC secondary antibody while microtubules were visualized with a mouse monoclonal antibody to alpha-tubulin conjugated to FITC (Figure 3A). The specificity of the antibody in immunofluorescence assays has been established by RNAi (Additional file 1: Figure S1B). From the asynchronously growing culture, we identified cells in prophase, metaphase, anaphase and telophase, and assayed for Mcm10 and alpha-tubulin localization (Figure 3A). We observed that the cells in prophase or prometaphase, with condensed chromatin and bi-polar centrosomes, were negative for Mcm10 signal (Figure 3A, top panel). In the same fields, we could observe interphase cells that were positive for Mcm10 signal, ruling out errors in our visualization method (arrows in fields 1, 2 and 3 point to prophase cells while other cells in same fields are in interphase). As previously reported, not all interphase cells are positive for Mcm10 signal and presumably these are the cells in early G_1 _phase. Similarly, cells in metaphase, identified by the presence of equatorial plate and polar microtubules, were also negative for Mcm10 signal (Figure 3A, middle panel). Cells in anaphase and telophase identified by segregated sister chromatids and shortened kinetochore microtubules, were also negative for Mcm10 signal (Figure 3A, third and bottom panel). To confirm that the loss of detection of Mcm10 is not due to the protein becoming soluble during mitosis and that our immunofluorescence protocol can detect soluble proteins during mitosis, we have compared the levels of Mcm10 and cyclin B in nocodazole blocked cells. HeLa cells arrested in prometaphase (marked by arrowheads) were negative for Mcm10 signal but retained the cyclin B signal, establishing the accuracy of our immunofluorescence assay (Additional file 3: Figure S2A). Therefore, our results demonstrate that the levels of Mcm10 protein are significantly reduced during mitosis.

**Figure 4:**
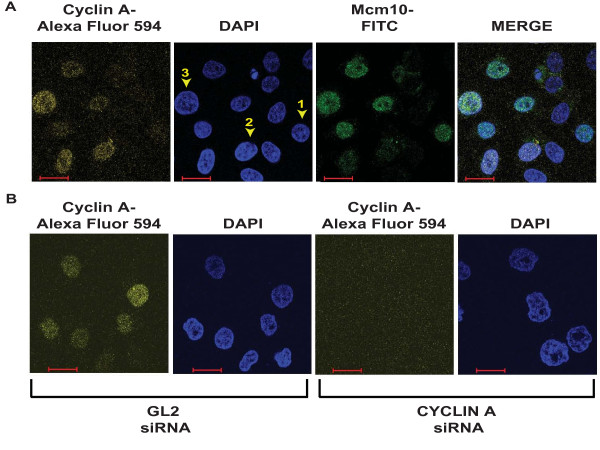
**Mcm10 is present in most cells that were positive for cyclin A**. (A) A field of asynchronously growing HeLa cells visualized for Mcm10 and cyclin A. Mcm10 was visualized by a rabbit anti-Mcm10 antibody in conjugation with anti-rabbit FITC antibody (third panel) while cyclin A was visualized with mouse anti-cyclin A antibody in conjugation with anti-mouse Alexa 594 antibody (first panel). DNA was stained with DAPI (second panel) while the right panel is a merge of images obtained from FITC, Alexa 594 and DAPI immunofluorescence. Cell 1 is positive for FITC and negative for Alexa 594 signal while cell 2 is positive for Alexa 594 but negative for FITC signal and cell 3 is positive for both demonstrating that there is no bleed-through of fluorescent signals. (B) RNAi confirms the cyclin A immunofluorescence signal while Mcm10 signal has been authenticated previously [11]. HeLa cells were transfected with *GL2 *or *CYCLIN A *siRNA oligos and later processed for immunofluorescence with the mouse anti-cyclin A antibody. The scale bar is 20 microns.

It is known that nocodazole interferes with the polymerization of microtubules and cells treated with nocodazole enter mitosis but cannot form metaphase spindles thereby activating the spindle assembly checkpoint which causes an arrest in prometaphase. As explained earlier in Figure 1, HeLa cells were treated with colchicine, nocodazole, taxol or vincristine for 15 h to obtain cells arrested in prometaphase. Incubation with any of these drugs resulted in disorganized microtubules and condensed chromosomes, confirming a M-phase block (Figure 3C). Prometaphase cells have been marked by arrowheads and we observed that these cells were negative for Mcm10 signal confirming that Mcm10 degradation has been initiated at this stage of mitosis. We determined the levels of endogenous Mcm10 and cyclin A in an asynchronously growing culture of HeLa cells by immunofluorescence using a rabbit anti-Mcm10 antibody, Ab (N), and mouse anti-cyclin A antibody respectively (Figure 4A). Utilizing the same antibodies, we have demonstrated that the cyclin A levels begin to increase in the S phase and it is degraded in the M-phase (Additional file 4: Figure S3). We observed that Mcm10 was present till the G_2 _phase as almost all the cells (94%) that retained cyclin A signal also stained positive for Mcm10 (Figure 4A). Combining the above results, we establish that Mcm10 is present till the G_2 _phase and is degraded around the G_2_/M boundary.

#### Live-cell imaging demonstrates that Mcm10 is absent during the M-phase

**Figure 5:**
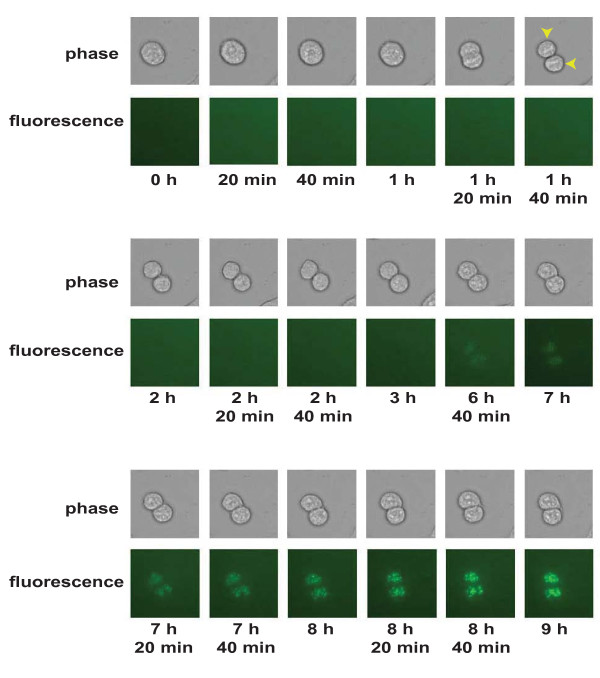
**Time-lapse imaging analysis of asynchronous culture of 293 cells expressing EGFP-Mcm10**. 293 cells were transfected with pEGFPC3-Mcm10 and 24 h after transfection, cells were placed on a live cell imaging stage (37°C with 5% CO_2_), and images were captured at 20 min intervals with a 10× objective of a Zeiss Observer Z1 inverted fluorescent microscope using an AxioCam HRm digital CCD camera. Some of the representative images have been shown along with the time elapsed since the start of imaging. Top rows are phase-contrast images while the bottom rows are corresponding EGFP fluorescent images in dark field. The two arrows indicate the daughter cells after cytokinesis at 1 h 40 min.

An alternate approach to address the levels of Mcm10 during mitosis would be to evaluate the cycling of Mcm10 in live cells. Since it has been reported that Mcm10 protein expression is not mainly regulated at the transcriptional level, we expressed EGFP-tagged Mcm10 from cytomegalovirus immediate early promoter [9]. HeLa cells were transfected with pEGFP C3-Mcm10 and two days after transfection, we observed a 140 kDa band with Mcm10 immunoblot, confirming the expression of EGFP-tagged Mcm10 (Additional file 5: Figure S4A). HeLa cells transfected with blank pEGFP-C3 vector displayed cytoplasmic EGFP signal while EGFP expressed in fusion with Mcm10 was guided to the nucleus (Additional file 5: Figure S4B). We evaluated the EGFP signal during mitosis, when the cells appear rounded in phase-contrast microscope. We expressed the EGFP-tagged full-length Mcm10 in 293 cells and tracked the EGFP signal and cell division of 293 cells (Figure 5). The M-phase is known to last around 1-1.5 h and as shown in Figure 5, we located a cell that is in mitosis and undergoes cytokinesis after 1 h 40 min (the two daughter cells have been marked by arrows). We observed that the cell was negative for Mcm10 during mitosis but the daughter cells accumulated Mcm10 after 8 h, during the G_1 _phase of the cell cycle. The overexpression of Mcm10 leads to a cell-cycle block during interphase and therefore the progression of cells from G_2 _to M could not be observed.

We have reported that the 61 aa ZF motif (783-843aa) of Mcm10 is sufficient for M phase proteolysis and therefore we expressed EGFP-tagged ZF motif of Mcm10 and tracked the EGFP signal and cell division of HeLa cells by fluorescence and phase-contrast microscopy respectively as the cells progressed through mitosis. As shown in Figure S4C (Additional file 5), we located a mitotic cell that undergoes cytokines after 1 h 20 min (the two daughter cells have been marked by arrows). We observed that the cell was negative for Mcm10 during mitosis but the daughter cells accumulated Mcm10 after around 5 h. Therefore, utilizing a different cell-line, we establish that the full-length Mcm10 is low during mitosis. Summing up, using this antibody-independent approach, we demonstrated that the Mcm10 levels are low during mitosis.

### APC is not required for cell cycle degradation of Mcm10

**Figure 6:**
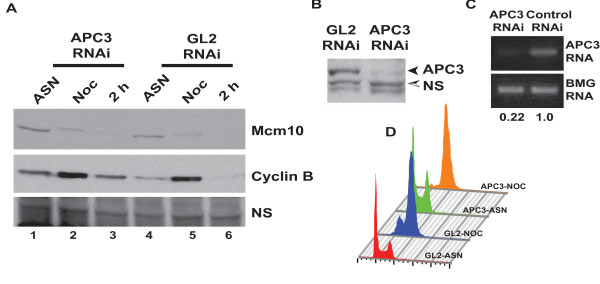
**Cell cycle degradation of Mcm10 is independent of APC**. (A) HeLa cells were transfected on three consecutive days with *APC3 *or control *GL2 *siRNA. After the third transfection, the cells were arrested with nocodazole for 15 h and were harvested after release from nocodazole (Noc) for analysis of Mcm10 protein, along with asynchronous cells (ASN). Samples were also taken 2 h after release from nocodazole (2 h) (B) The decrease of APC3 protein after RNAi was confirmed by immunoblotting with anti-APC3 antibody. NS points to a non-specific band that displays equal protein loading in different lanes. (C) The decrease of *APC3 *mRNA was confirmed by RT-PCR and the numbers indicate the relative intensity of the mRNA. (D) Flow cytometry of propidium iodide-stained DNA of HeLa cells, as described in (A) confirms a M-phase block and release after siRNA depletion. The observed mobility of endogenous APC3 was 73 kDa.

APC/C or cyclosome is a complex of many proteins that functions as an E3 ubiquitin ligase during mitosis. Since Mcm10 is low in the M-phase, there is a distinct possibility that APC is involved in degradation of Mcm10. Notably, Mcm10 contains destruction box sequence (REQLAYLES) and a KEN box, motifs that are required for recognition of substrates by APC [12]. To test whether APC mediates the M-phase degradation of Mcm10, we silenced APC3 subunit of APC, which would effectively debilitate its ubiquitination ability. HeLa cells transfected with *APC3 *siRNA for three consecutive days were blocked in the M-phase with nocodazole for 15 h and subsequently released into nocodazole free medium. RNAi against *APC3 *decreased the target protein and mRNA but that did not increase the levels of Mcm10 in nocodazole blocked cells (Figure 6). RNAi effectively inhibited the APC activity as evidenced by increase in the levels of cyclin B (compare lanes 3 with 6 in Figure 6A). This strongly suggests that APC is not involved in the cell-cycle degradation of Mcm10. We have assayed the Mcm10 levels in asynchronous populations of cells transfected with *APC3*, *CUL1*, *CDH1*, *CDC20*, *BETA-TRCP *and *FBXW7 *or control *GL2 *siRNA and observed that only minor variations were observed in Mcm10 levels (Additional file 2: Figure S5). We have previously reported that UV-irradiation specifically proteolyses Mcm10 and we have identified the E3 ubiquitin ligase mediates the UV-triggered and M-phase proteolysis of Mcm10 [11].

### APC recognition motifs are not required for cell cycle degradation of Mcm10

**Figure 7:**
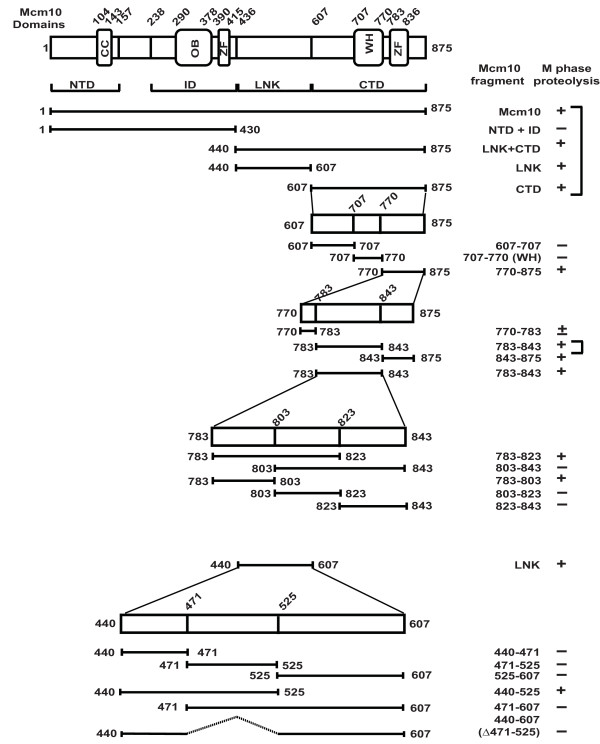
**Schematic representation of Mcm10 and the fragments cloned into the pMX-puro vector**. NTD, ID, LNK and CTD refer to N-terminal, inner, linker and C-terminal domains. OB, ZF and WH refer to oligonucleotide binding, zinc finger and winged helix motifs, respectively. The locations of Mcm10 fragments and the proteolysis during M-phase have been summarized based on the results obtained from Figure 8 and 9. Previously published results have been marked with ']'.

We have previously utilized stable U2OS cells expressing HA-tagged Mcm10 (utilizing the pMX-retroviral vector that is based on the moloney murine leukemia virus) to determine the segments of Mcm10 that are essential for cell cycle-regulated degradation [11]. Full-length Mcm10 can be broadly divided into N-terminal (NTD), inner (ID), linker (LNK) and C-terminal (CTD) domains (Figure 7). A coiled-coil motif is present within the N-terminus of Mcm10, which is required for homodimerization. The inner and C-terminal domains, which contain zinc finger (ZF) and winged helix (WH) motifs, bind to single- and double-stranded DNA, and the p180 subunit of DNA polymerase-alpha [13,14]. As reported previously, we observed that the HA-tagged full length Mcm10 protein behaves like the endogenous protein [11]. U2OS cells expressing HA-tagged full length Mcm10 were blocked with nocodazole, released and harvested at regular intervals in order to collect cells in different phases of the cell cycle. Endogenous Mcm10 levels were low in M phase and increased around 8 h after release from nocodazole block (Figure 2B). HA-tagged full-length Mcm10 showed a degradation pattern similar to that of the endogenous protein, which demonstrates that our assay for evaluating the degradation of different regions of Mcm10 is accurate.

We observed that the NTD+ID domain of Mcm10, which contains the KEN box, was resistant to cell cycle-regulated degradation. In the present study, we tested whether the WH motif, that contains the destruction box, is proteolyzed during the M-phase. Stable U2OS cells expressing WH motif of Mcm10 (707-770 aa) was blocked with nocodazole, which arrested the cells in M-phase. The obtained mitotic cells were released from the block and harvested at regular intervals in order to collect cells in different phases of the cell cycle. The flow cytometry profile of propidium iodide-stained DNA and the levels of cyclin B demonstrate a block in M-phase and subsequent progression through the cell cycle (Figure 2D). As noted previously, Mcm10 was absent in nocodazole-released cells, demonstrating the natural proteolysis of Mcm10 in M phase which began to increase after 8 h, displaying the natural cycling of Mcm10 levels (Figure 2C). Full-length Mcm10 expressed from the retroviral vector showed a degradation pattern similar to that of the endogenous protein, validating our assay for evaluating the cell cycle-regulated degradation of Mcm10. We observed that the WH motif fragment was resistant to cell cycle-regulated degradation. We confirmed that the nuclear localization signal expressed in fusion with the WH motif steered it to the nucleus and therefore change in cellular localization is not the reason for resistance to proteolysis (Figure 2E). We have previously reported that the LNK domain which does not have either the KEN box or destruction box is proteolyzed as cells pass through M-phase. Therefore, we conclude that the KEN and destruction box of Mcm10 are neither essential nor sufficient for its M-phase proteolysis.

### Mcm10 proteolysis utilizes independent non-overlapping signals

**Figure 8:**
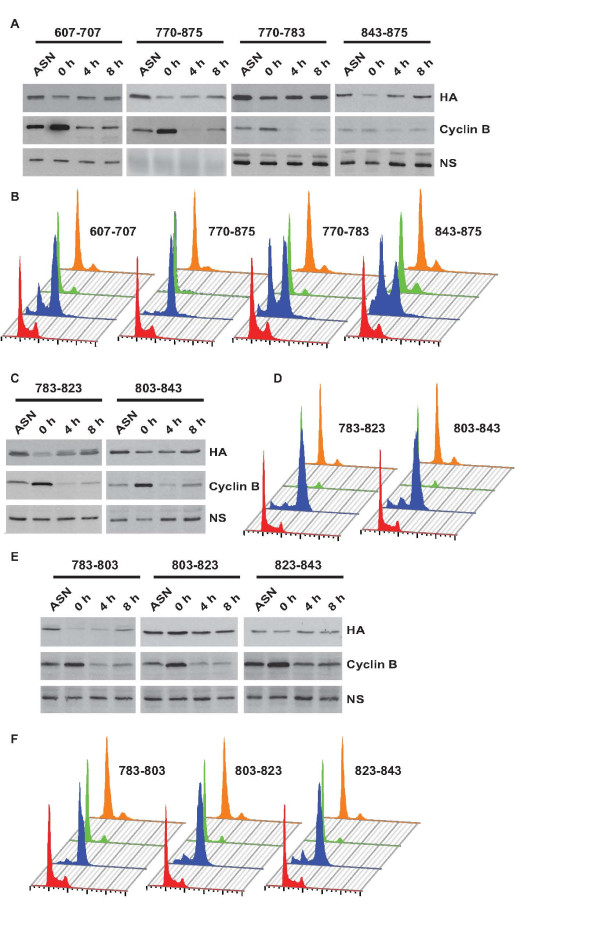
**Independent non-overlapping regions are sufficient for Mcm10 proteolysis during M-phase**. U2OS cells expressing different regions of Mcm10 were arrested in M-phase using nocodazole (0 h) and then harvested at the indicated time-points after release into drug-free medium. The proteolysis of different regions of Mcm10 evaluated after release from nocodazole by immunoblotting with an anti-HA antibody has been shown in parts (A), (C) and (E) and the flow cytometry of propidium iodide-stained DNA of stable cell-line expressing the corresponding fragment has been shown in parts (B), (D) and (F) respectively. ASN refers to samples from asynchronously growing cells while NS points to a non-specific band that displays equal protein loading. The observed mobility of fragments of Mcm10 (fused to GFP and HA epitope tags) was as follows: 607-707: 52 kDa; 770-875: 52 kDa; 770-783: 42 kDa; 843-875: 42 kDa; 783-823: 44 kDa; 803-843: 44 kDa; 783-803: 40 kDa; 803-823: 44 kDa; 823-843: 43 kDa.

**Figure 9:**
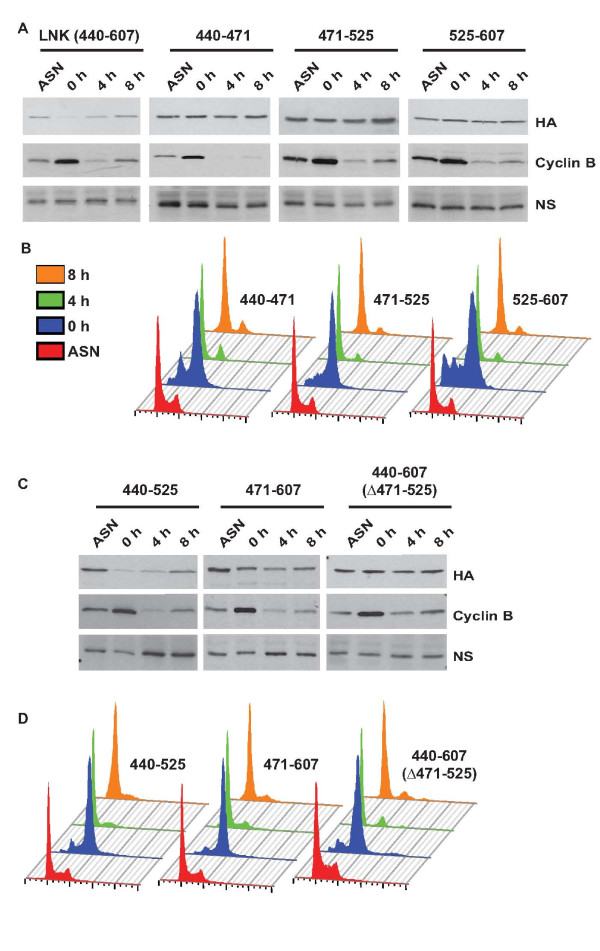
**Residues 440-525 within the Linker domain are required for Mcm10 proteolysis during M-phase**. U2OS cells expressing different regions of Mcm10 were arrested in M-phase using nocodazole (0 h) and then harvested at the indicated time-points after release into drug-free medium. The proteolysis of different regions of Mcm10 evaluated after release from nocodazole by immunoblotting with an anti-HA antibody has been shown in parts (A) and (C) and the flow cytometry of propidium iodide-stained DNA of stable cell-line expressing the corresponding fragment has been shown in parts (B) and (D) respectively. ASN refers to samples from asynchronously growing cells while NS points to a non-specific band that displays equal protein loading. The observed mobility of fragments of Mcm10 (fused to GFP and HA epitope tags) was as follows: LNK domain (440-607): 55 kDa; 440-471: 44 kDa; 471-525: 46 kDa; 525-607: 49 kDa; 440-525: 50 kDa; 471-607: 53 kDa; 440-607 (471-525): 53 kDa.

To identify the domains required for Mcm10 proteolysis during M-phase, we expressed different regions of Mcm10 and assayed their stability. As noted previously, the NTD+ID domain was resistant to cell cycle-regulated degradation, but the LNK and CTD domains were proteolyzed in M phase (Figure 8A). Though the ZF motif was sufficient for M phase proteolysis of Mcm10, the CTD domain lacking the ZF motif was also degraded in M phase, signifying that though ZF motif is sufficient, it is not essential for M phase proteolysis of Mcm10 (Figure 9A). We now wanted to identify the degron that is essential for degradation.

We further divided the CTD (607-875 aa) into three fragments 607-707 aa, 707-770 aa (WH motif) and 770-875 aa. We observed that the 607-707 aa and 707-770 aa fragment were resistant to proteolysis (Figure 8A, panel 1 and results of WH motif in Figure 2C). However, the 770-875 aa fragment decreased in M-phase, demonstrating that the signal for degradation lies in this fragment. In order to identify the minimum sequence required for Mcm10 degradation, we further divided the 770-875 aa fragment into three parts: 770-783 aa, 783-843 aa and 843-875 aa. 770-783 aa fragment was partially stable indicating that there could be an incomplete degron in this fragment (Figure 8A, panel 3). The 783-843 aa (ZF motif) and 843-875 aa decreased in M-phase demonstrating that there are non-overlapping regions that are sufficient for Mcm10 proteolysis (Figure 8A, panel 4 and results of ZF motif in [11]). The ZF motif was divided into 3 segments each of 20 amino acids: 783-803 aa, 803-823 aa and 823-843 aa. We observed that 783-803 aa region was essential and sufficient for degradation while 803-823 aa and 823-843 aa were not proteolyzed (Figure 8C and 8E). Therefore, we have narrowed to two regions that contain the degron: a 20 aa region (783-803 aa) within the ZF motif and C-terminal end of Mcm10 (843-875 aa).

Another independent region that is sufficient for Mcm10 downregulation is the linker domain (440-607 aa). We divided the linker domain into three parts: 440-471 aa, 471-525 aa and 525-607 aa. We observed that though the linker domain is decreased during M-phase, none of the three constituent fragments were proteolyzed (Figure 9A). However, when we combined the 440-471 aa and 471-525 aa fragments, the degradation ability was restored (Figure 9C, panel 1). Combining 440-471 aa with 525-607 aa or combing 471-525 aa with 525-607 aa did not result in M-phase degradation (Figure 9C, panel 2 and 3). Therefore, the degron in the linker domain lies within the stretch of 440-525 aa. Hence, it seems that Mcm10 degradation is mediated by non-overlapping regions, apparently to ensure downregulation of Mcm10 even if proteolysis at any one region is somehow blocked. There is no apparent sequence similarity between the three minimal regions that are competent for M-phase proteolysis: 440-525 aa, 783-803 aa and 843-875 aa. This would suggest that the ubiquitination machinery is adaptable to recognize multiple sequences to ensure Mcm10 proteolysis. Recognition of many biological targets is defined by similar 2-D structures rather than primary sequence and that possibility cannot be ruled for recognition of different domains of Mcm10.

## Discussion and Conclusions

We have observed that the Mcm10 levels are significantly reduced in nocodazole blocked cells. This is in contrast to the previous report where on the basis of estimation of Mcm10 levels in asynchronous and nocodazole released cells by immunoblots, the authors inferred that Mcm10 degradation begins after metaphase [9]. It is possible that the Mcm10 protein observed after nocodazole treatment was contributed by cells trapped at G_2_/M boundary. The stabilization of Mcm10 by MG132 in nocodazole released cells observed by them is likely due to non-degradation of Mcm10 in these cells. It has been suggested that a destruction box sequence (719-727 aa) could be recognized by the anaphase-promoting complex for ubiquitination. Additionally, Mcm10 also contains a KEN box sequence (71-73 aa) that is present in targets ubiquitinated by APC. However, the NTD domain, which contains the KEN box is not degraded during M-phase [11]. Similarly, the WH motif which contains the destruction box is stable during mitosis (Figure 2C). However, the linker region and ZF motif, which do not have either destruction or KEN box, are proteolyzed during M-phase. In summation, the above data would suggest that the APC recognition motifs are not required for M-phase degradation of Mcm10. In this study, we have also demonstrated that inhibition of APC does not block cell-cycle degradation of Mcm10.

This study has discovered at least three independent regions of Mcm10 that are sufficient for proteolysis during M phase. Since the non-overlapping regions of Mcm10 sufficient for M-phase proteolysis did not display sequence similarity, it suggests of a limited degeneracy of the E3 ligase to allow for recognition of multiple substrates. CRL4 is known to mediate the ubiquitination of almost two dozen substrates, including Cdt1, PCNA and histones that do not share sequence similarity [15]. It is widely believed that the proteolysis of Mcm10 during mitosis is a mechanism to prevent aberrant initiation of replication and therefore it is possible that utilization of independent regions is a means to ensure downregulation of Mcm10 even if proteolysis at any one region is somehow blocked. The cloning of stable mutants of full length Mcm10 would help us evaluate the effect of stable Mcm10 on cell-cycle progression and genomic stability. It is believed that the proteolysis of Mcm10 during mitosis is a vital mechanism to prevent aberrant initiation of replication and the present study describes the regulation of Mcm10 during this phase of the cell-cycle.

## Methods

### Cell culture, chemicals, antibodies, cell synchronization and FACS analysis

Cell lines were maintained in DMEM supplemented with fetal bovine serum and antibiotics. Specific chemicals and antibodies used in this study are mentioned in the supplementary methods (Additional file 6). HeLa cells were transfected with specific siRNA oligos consecutively for three days and were blocked with 40 ng/ml nocodazole for 15 h. Later, cells were released in drug-free medium and cells arrested in the M-phase were collected by 'mitotic-shake off'. The obtained mitotic cells were then re-plated in drug-free medium and harvested till 12 h to collect cells in different phases of the cell cycle. HeLa and U2OS cells were incubated with 40 ng/ml or 0.10 μg/ml nocodazole, 0.05 μg/ml or 0.2 μg/ml vincristine, 0.15 μg/ml or 0.5 μg/ml colchicine or 0.1 μg/ml or 0.3 μg/ml taxol for 15 h or 16 h respectively to obtain mitotic cells. For cell cycle analysis, the cells were fixed with 70% ethanol after washing with 1× PBS. Subsequently, the cell pellet was resuspended in 1× PBS with 0.1% Triton X-100, 20 μg/ml RNase and 70 μg/ml propidium iodide and the flow cytometry was performed.The flow cytometry data was acquired on Becton Dickinson FACS Calibur machine by Cell Quest Pro software. Cell cycle analysis was done by Dean/Jett/Fox method of FlowJo software.

### Antibodies for immunoblotting and immunofluorescence

Anti-human Mcm10 antibody was produced using recombinant His-tagged Mcm10 (cloned in pET28a vector), purified on nickel-NTA column (Qiagen).Rabbits were injected with recombinant protein along with complete Freund's adjuvant to obtain Mcm10 Ab (N) and Ab (FL). Anti-Mcm10 sera was affinity-purified using Mcm10 conjugated sepharose column. Rabbit polyclonal anti-cyclin B1 (Cat. No. Sc-752) was purchased from Santa Cruz Biotechnology Mouse monoclonal anti-APC3 (Cat. No. ab10538) and anti-alpha tubulin-FITC (Cat. No. ab64503) were purchased from Abcam.Mouse monoclonal anti-cyclin A (Cat. No. CS-4656) was obtained from Cell Signaling Technology.Mouse monoclonal anti-HA (Cat.No. H3663) was obtained from Sigma. Polyclonal goat anti-rabbit HRP (Cat.No. P0448), polyclonal rabbit anti-mouse HRP (Cat.No. P0161) and polyclonal swine anti-rabbit TRITC (Cat No.R0156) were obtained from Dako. Alexa Fluor 488 goat anti-rabbit IgG (Cat.No. A11008) and Alexa Fluor 594 goat anti-mouse IgG (Cat.No. A110050) were obtained from Molecular Probes, Invitrogen. For western blotting, cells were harvested in 1× SDS sample buffer. Equal amount of protein was separated on SDS-polyacrylamide gels and then transferred onto nitrocellulose membranes. Finally the results were assayed using the Enhanced Chemiluminescence method.

### RNAi silencing and reverse-transcriptase PCR

Silencing of genes was done by transfecting specific 40-80 nM siRNA duplex on three consecutive days and cells were harvested 24 h post the last transfection.The levels of protein and mRNA were evaluated by immunoblotting and reverse-transcriptase PCR respectively. For reverse-transcriptase PCR, RNA was extracted using TRIzol method and 0.25-1 μg RNA was used for cDNA synthesis. The primers used for PCR are described in the supplementary methods (Additional file 6)

### Plasmid construction, transfection, immunoblotting and immunofluorescence

Full-length Mcm10 was subcloned into BglII and SalI sites of pEGFP-C3 vector, which carries the GFP sequence at the N-terminal of the vector. Similarly, Mcm10 cDNA was digested with either BglII and EcoRI or BglII and MfeI and cloned in BamHI and EcoRI sites of pMX-puro-NLS-HA vector. The sequences of the cloning primers are provided in the supplementary methods (Additional file 6). Stable U2OS cells expressing Mcm10 and its fragments were generated as described previously [11]. Cells of almost equal confluency were lysed in proportionate volumes of Laemmli buffer for immunoblotting. To demonstrate equal protein loading in each lane, immunoblotting was performed and a nonspecific protein band was displayed. For indirect immunofluorescence studies, HeLa cells grown on glass coverslips in DMEM supplemented with 10% fetal bovine serum (FBS) were fixed with 4% formaldehyde in PBS for 10 min and then permeabilized with 0.2% Triton X-100 in PBS for 5 min. Later, the cells were blocked with 10% FBS in PBS with 0.1% Tween-20. The cells were then stained with primary antibody (1:100 or 1:500 dilution) for an hour followed by either fluorescein isothiocyanate, Alexa-488 or Alexa-594-conjugated anti-rabbit or anti-mouse antibody (1:500 dilution) at room temperature. The coverslips were then mounted with a mounting reagent with DAPI and viewed under the Nikon TE2000-S inverted fluorescence microscope. Images were captured on Evolution VF (Media Cybernetics) 12-bit color digital camera using the 'Q capture Pro' software and contrast enhancements were identically done for all the images of a particular antibody/protein in an experiment. Some images were captured using the Zeiss LSM 510 confocal microscope and viewed using the Zeiss LSM Image Browser Version 4,2,0,121 software.

## Authors' contributions

MK carried out gene silencing and time course experiments. AK and AS carried out time course experiments and also along with MMK determined the domains essential for Mcm10 degradation. All authors read and approved the final manuscript.

## Supplementary Material

Additional file 1 **Figure S1: RNA interference authenticates the Mcm10 protein in immunoblotting and immunofluorescence assays**. (A, B) HeLa cells were transfected with *GL2 *or *MCM10 *siRNA and subsequently processed for immunoblotting with anti-Mcm10 antibodies (Ab [N] and Ab [FL]) or immunofluorescence with anti-Mcm10 antibody (Ab [N]). NS points to a non-specific band that displays equal protein loading. Click here for file

Additional file 2 **Figure S5: RNA interference authenticates the Mcm10 protein in immunoblotting assays**. (A) HeLa cells were transfected with GL2 or MCM10 siRNA and subsequently processed for immunoblotting with anti-Mcm10 antibodies (Ab [N] and Ab [FL]) to display the whole gel. The black and shaded arrow head points to the Mcm10 and a cross-reactive band respectively while NS refers to a non-specific band that displays equal protein loading. (B) Mcm10 levels after depletion of APC3, Cul1, Cdh1 and Cdc20. HeLa cells were transfected on three consecutive days with APC3, CUL1, CDH1, CDC20 or control GL2 siRNA and after the third transfection, asynchronous cells (ASN) were harvested for analysis of Mcm10 protein. NS points to a non-specific band that displays equal protein loading. (C) Mcm10 levels after depletion of beta-TRCP and FBXW7. HeLa cells were transfected on three consecutive days with BETA-TRCP, FBXW7 or control GL2 siRNA. After the third transfection, the cells were arrested with nocodazole for 15 h and were harvested after release from nocodazole (Noc) for analysis of Mcm10 protein, along with asynchronous cells (ASN). (D) The decrease of BETA-TRCP and FBXW7 mRNA was confirmed by RT-PCR. The numbers indicate the relative intensity of specific mRNA. Click here for file

Additional file 3 **Figure S2: Cyclin B is detectable in nocodazole blocked cells**. (A) Nocodazole blocked HeLa cells were visualized for Mcm10 and cyclin B. Mcm10 was visualized by anti-Mcm10 antibody in conjugation with anti-rabbit Alexa 488 antibody (top-right panel) while cyclin B was visualized with anti-cyclin B antibody in conjugation with anti-rabbit Alexa 488 antibody (bottom-right panel). DNA was stained with DAPI (left panel). HeLa cells that are blocked in mitosis have been marked by arrowheads. (B) HeLa cells were harvested after release from (Noc) and treated with MG132 as indicated and immunoblotted with anti-Mcm10 antibody (top panel). ASN refers to samples from asynchronously growing cells not treated with nocodazole while NS points to a non-specific band that displays equal protein loading. U2OS cells expressing HA-tagged full length Mcm10 (second panel) or HA-tagged linker domain of Mcm10 (third panel) were treated similarly. Since MG132 interfered with the nocodazole block in U2OS cells, the total population of cells after treatment with nocodazole and MG132 was loaded in second and third panel. The observed mobility of the linker domain of Mcm10 (fused to GFP and HA epitope tags) was approximately 55 kDa. Click here for file

Additional file 4 **Figure S3: Immunoblotting and immunofluorescence assays demonstrate decrease in cyclin A protein as cells enter mitosis**. (A) HeLa cells were synchronized at G_1_/S transition with hydroxyurea and later released and samples were collected at 4 h intervals and processed for immunoblotting and immunofluorescence. Cyclin A was visualized with mouse anti-cyclin A antibody in conjugation with anti-mouse Alexa 594 antibody (right panel) while DNA was stained with DAPI (left panel). Representative fields of different time points have been shown. Note that Alexa 594 emission is visible as a red color after passing through the emission filter of Nikon TE2000-S inverted fluorescence microscope. (B) Levels of endogenous cyclin A and Mcm10 were evaluated by specific antibody. ASN refers to samples from asynchronously growing cells while NS points to a non-specific band that displays equal protein loading in different lanes. (C) Flow cytometry of propidium iodide-stained DNA of HeLa cells, as described in (A), shows a G_1_/S block and release. The colored key denotes cells obtained at different time-points and the FACS histogram shows peaks corresponding to different phases of the cell cycle. Click here for file

Additional file 5 **Figure S4: Expression of GFP signal in HeLa cells transfected with either pEGFPC3 or pEGFPC3-Mcm10**. (A) Transfected HeLa cells were harvested and analyzed by rabbit anti-Mcm10 antibody which identifies the mobility of the endogenous Mcm10 (*shaded arrowhead*) and exogenously expressed EGFP-Mcm10 (*black arrowhead*). (B) Two days after transfection, cells were placed on a live cell imaging stage (37°C with 5% CO2), and images were captured with a 40× objective of a Zeiss Observer Z1 inverted fluorescent microscope using an AxioCam HRm digital CCD camera. (C) Time-lapse imaging analysis of asynchronous culture of HeLa cells expressing EGFP-Mcm10. HeLa cells were transfected with pEGFPC3-ZF Mcm10 and 24 h after transfection, cells were placed on a live cell imaging stage (37°C with 5% CO_2_), and images were captured at 20 min intervals as described in Figure 4. Some of the representative images have been shown along with the time elapsed since the start of imaging. Top rows are phase-contrast images while the bottom rows are corresponding EGFP fluorescent images in dark field. The two arrows indicate the daughter cells after cytokinesis at 1 h 20 min. Click here for file

Additional file 6 **Supplementary Methods**. Click here for file
