# Supplementary figures and images for: Mcm10 proteolysis initiates before the onset of M-phase
Source: BMC Cell Biol. 2010 Oct 28;11:84. doi: 10.1186/1471-2121-11-84 (PMC2987893; doi:10.1186/1471-2121-11-84)

**Figure S1**

**A**

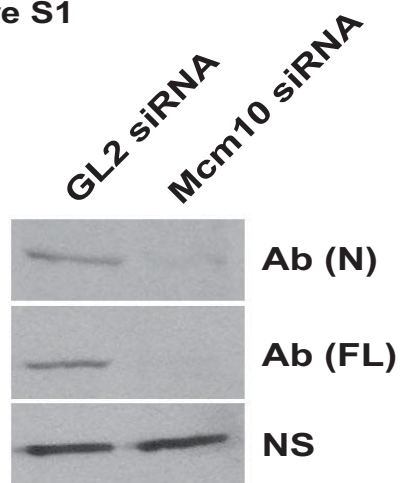

**B**

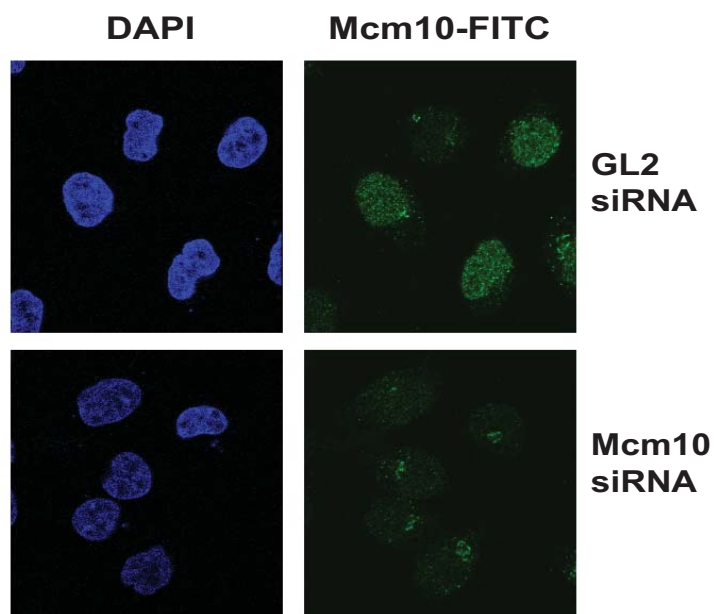

Supplement: Additional file 1 — Figure S1: RNA interference authenticates the Mcm10 protein in immunoblotting and immunofluorescence assays. (A, B) HeLa cells were transfected with GL2 or MCM10 siRNA and subsequently processed for immunoblotting with anti-Mcm10 antibodies (Ab [N] and Ab [FL]) or immunofluorescence with anti-Mcm10 antibody (Ab [N]). NS points to a non-specific band that displays equal protein loading. [file 1471-2121-11-84-S1.PDF]

Figure S5

A

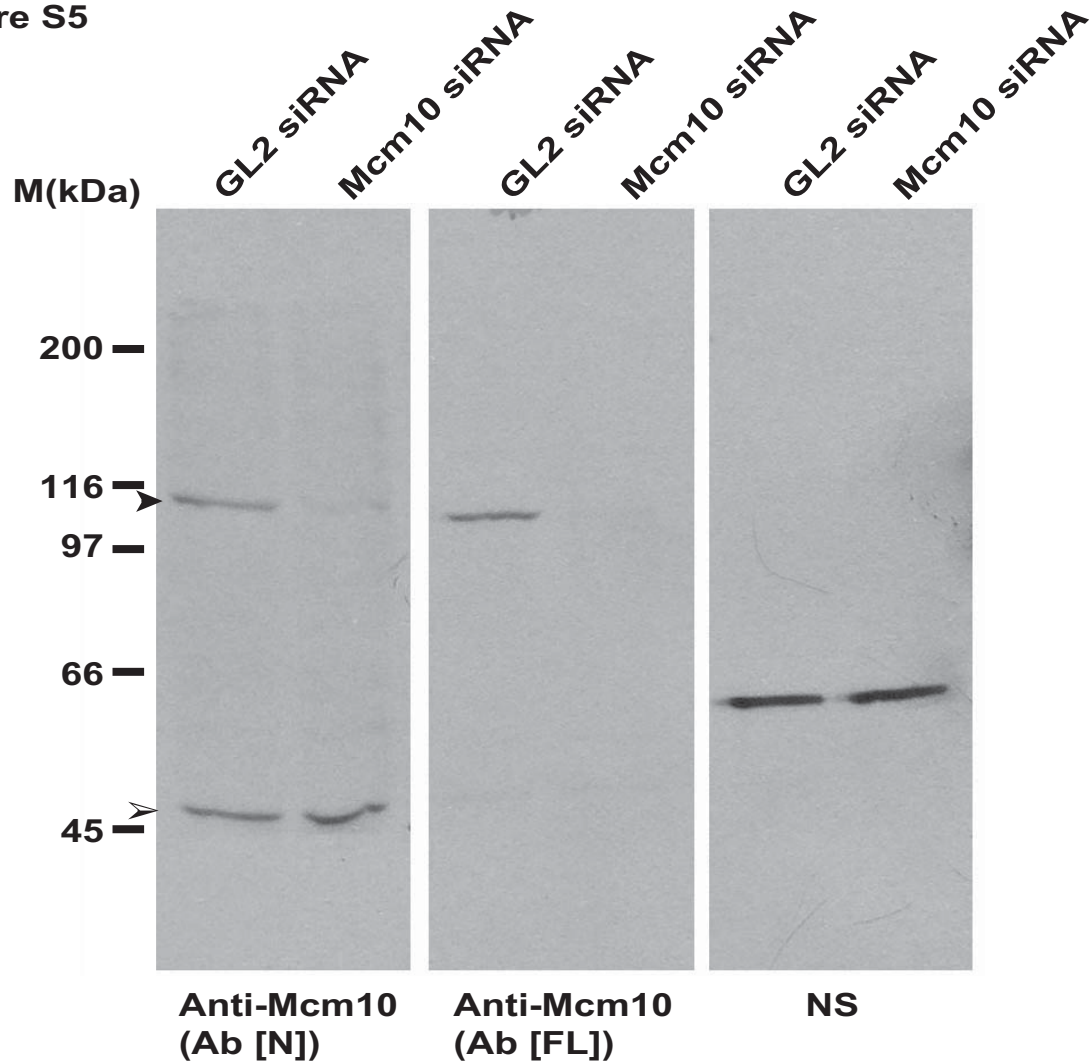

B

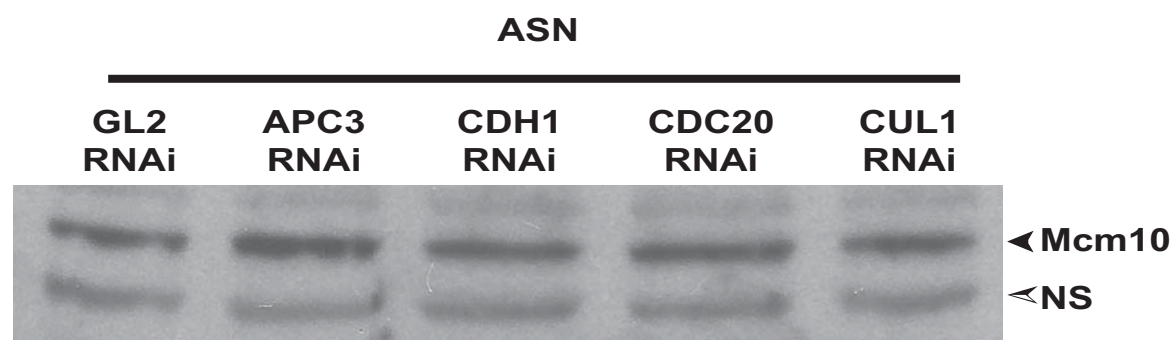

C

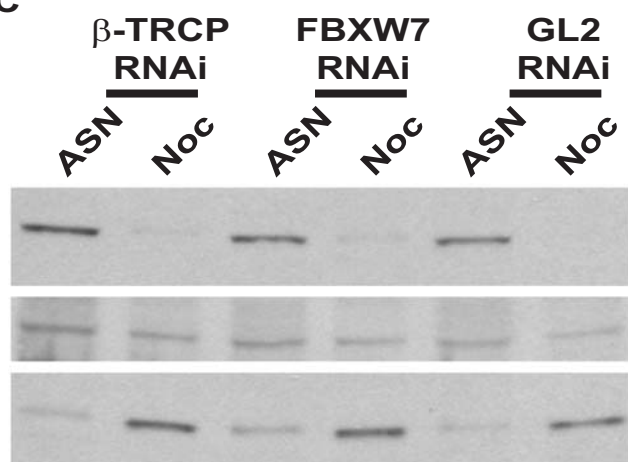

D

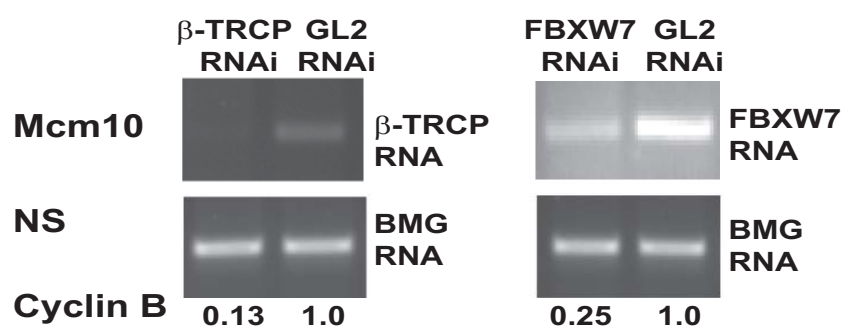

Supplement: Additional file 2 — Figure S5: RNA interference authenticates the Mcm10 protein in immunoblotting assays. (A) HeLa cells were transfected with GL2 or MCM10 siRNA and subsequently processed for immunoblotting with anti-Mcm10 antibodies (Ab [N] and Ab [FL]) to display the whole gel. The black and shaded arrow head points to the Mcm10 and a cross-reactive band respectively while NS refers to a non-specific band that displays equal protein loading. (B) Mcm10 levels after depletion of APC3, Cul1, Cdh1 and Cdc20. HeLa cells were transfected on three consecutive days with APC3, CUL1, CDH1, CDC20 or control GL2 siRNA and after the third transfection, asynchronous cells (ASN) were harvested for analysis of Mcm10 protein. NS points to a non-specific band that displays equal protein loading. (C) Mcm10 levels after depletion of beta-TRCP and FBXW7. HeLa cells were transfected on three consecutive days with BETA-TRCP, FBXW7 or control GL2 siRNA. After the third transfection, the cells were arrested with nocodazole for 15 h and were harvested after release from nocodazole (Noc) for analysis of Mcm10 protein, along with asynchronous cells (ASN). (D) The decrease of BETA-TRCP and FBXW7 mRNA was confirmed by RT-PCR. The numbers indicate the relative intensity of specific mRNA. [file 1471-2121-11-84-S2.PDF]

**Figure S2**

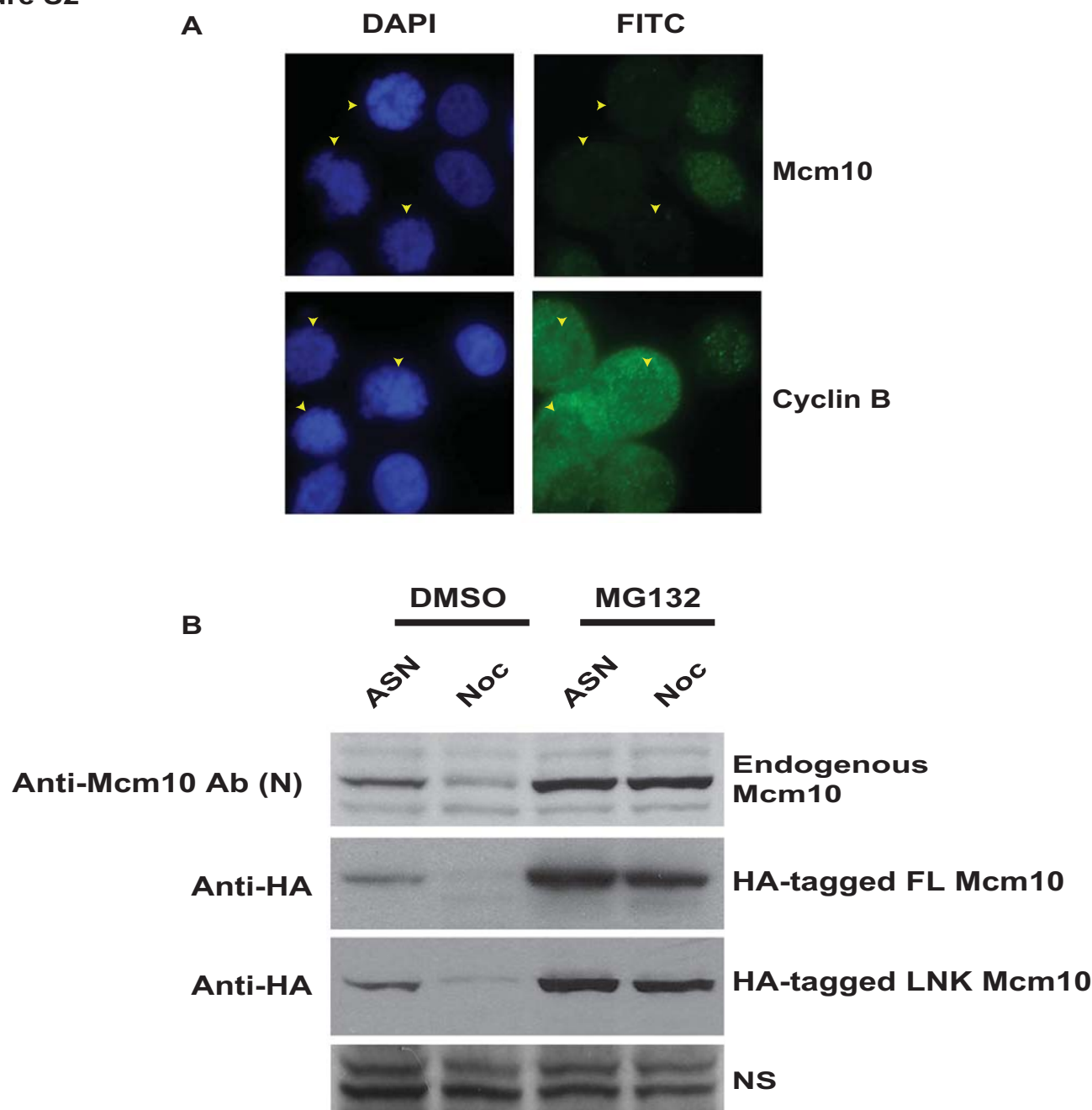

Supplement: Additional file 3 — Figure S2: Cyclin B is detectable in nocodazole blocked cells. (A) Nocodazole blocked HeLa cells were visualized for Mcm10 and cyclin B. Mcm10 was visualized by anti-Mcm10 antibody in conjugation with anti-rabbit Alexa 488 antibody (top-right panel) while cyclin B was visualized with anti-cyclin B antibody in conjugation with anti-rabbit Alexa 488 antibody (bottom-right panel). DNA was stained with DAPI (left panel). HeLa cells that are blocked in mitosis have been marked by arrowheads. (B) HeLa cells were harvested after release from (Noc) and treated with MG132 as indicated and immunoblotted with anti-Mcm10 antibody (top panel). ASN refers to samples from asynchronously growing cells not treated with nocodazole while NS points to a non-specific band that displays equal protein loading. U2OS cells expressing HA-tagged full length Mcm10 (second panel) or HA-tagged linker domain of Mcm10 (third panel) were treated similarly. Since MG132 interfered with the nocodazole block in U2OS cells, the total population of cells after treatment with nocodazole and MG132 was loaded in second and third panel. The observed mobility of the linker domain of Mcm10 (fused to GFP and HA epitope tags) was approximately 55 kDa. [file 1471-2121-11-84-S3.PDF]

Figure S3

A

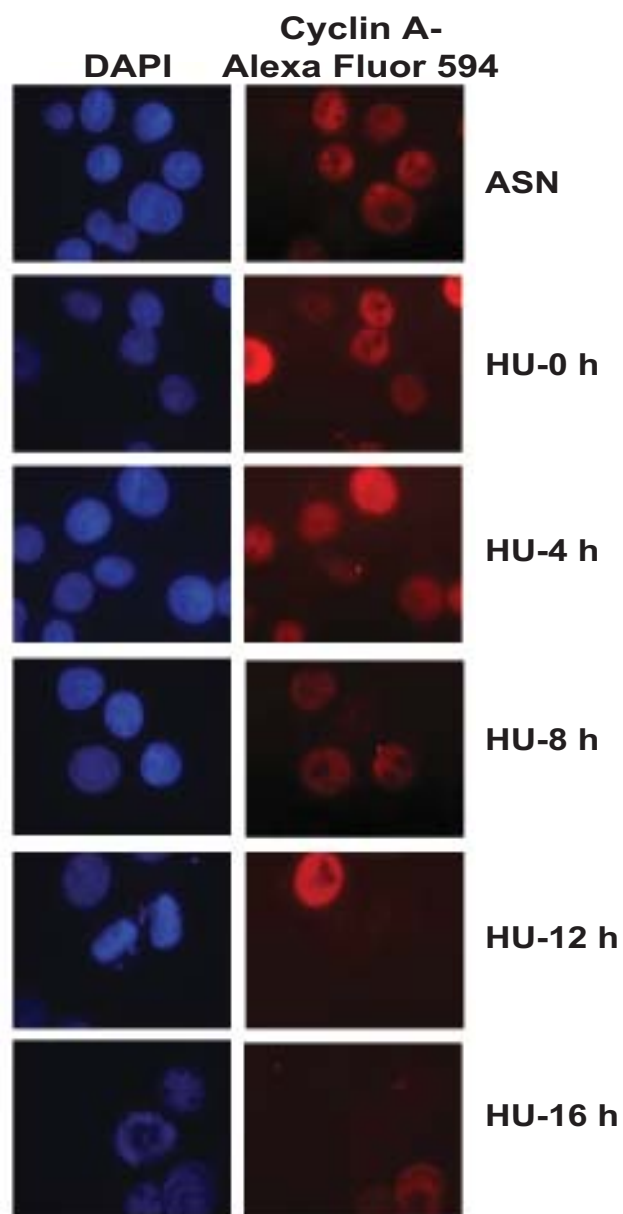

B

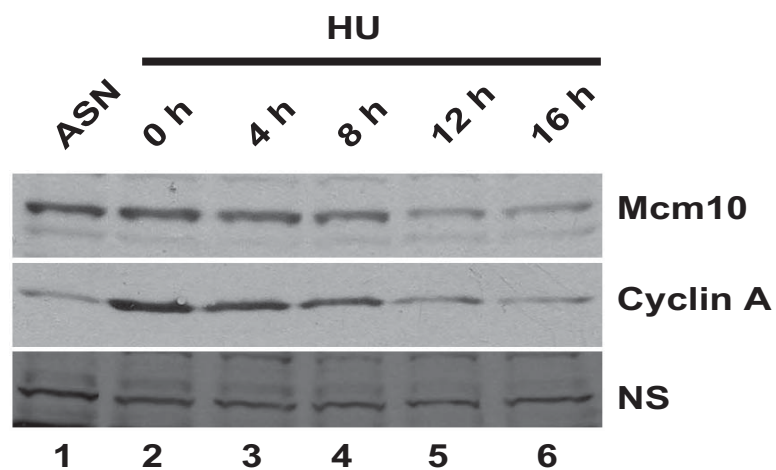

C

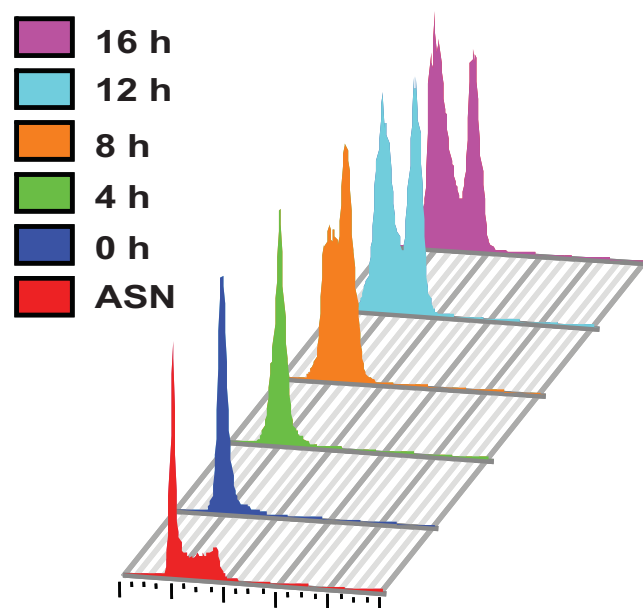

Supplement: Additional file 4 — Figure S3: Immunoblotting and immunofluorescence assays demonstrate decrease in cyclin A protein as cells enter mitosis. (A) HeLa cells were synchronized at G1/S transition with hydroxyurea and later released and samples were collected at 4 h intervals and processed for immunoblotting and immunofluorescence. Cyclin A was visualized with mouse anti-cyclin A antibody in conjugation with anti-mouse Alexa 594 antibody (right panel) while DNA was stained with DAPI (left panel). Representative fields of different time points have been shown. Note that Alexa 594 emission is visible as a red color after passing through the emission filter of Nikon TE2000-S inverted fluorescence microscope. (B) Levels of endogenous cyclin A and Mcm10 were evaluated by specific antibody. ASN refers to samples from asynchronously growing cells while NS points to a non-specific band that displays equal protein loading in different lanes. (C) Flow cytometry of propidium iodide-stained DNA of HeLa cells, as described in (A), shows a G1/S block and release. The colored key denotes cells obtained at different time-points and the FACS histogram shows peaks corresponding to different phases of the cell cycle. [file 1471-2121-11-84-S4.PDF]

Figure S4

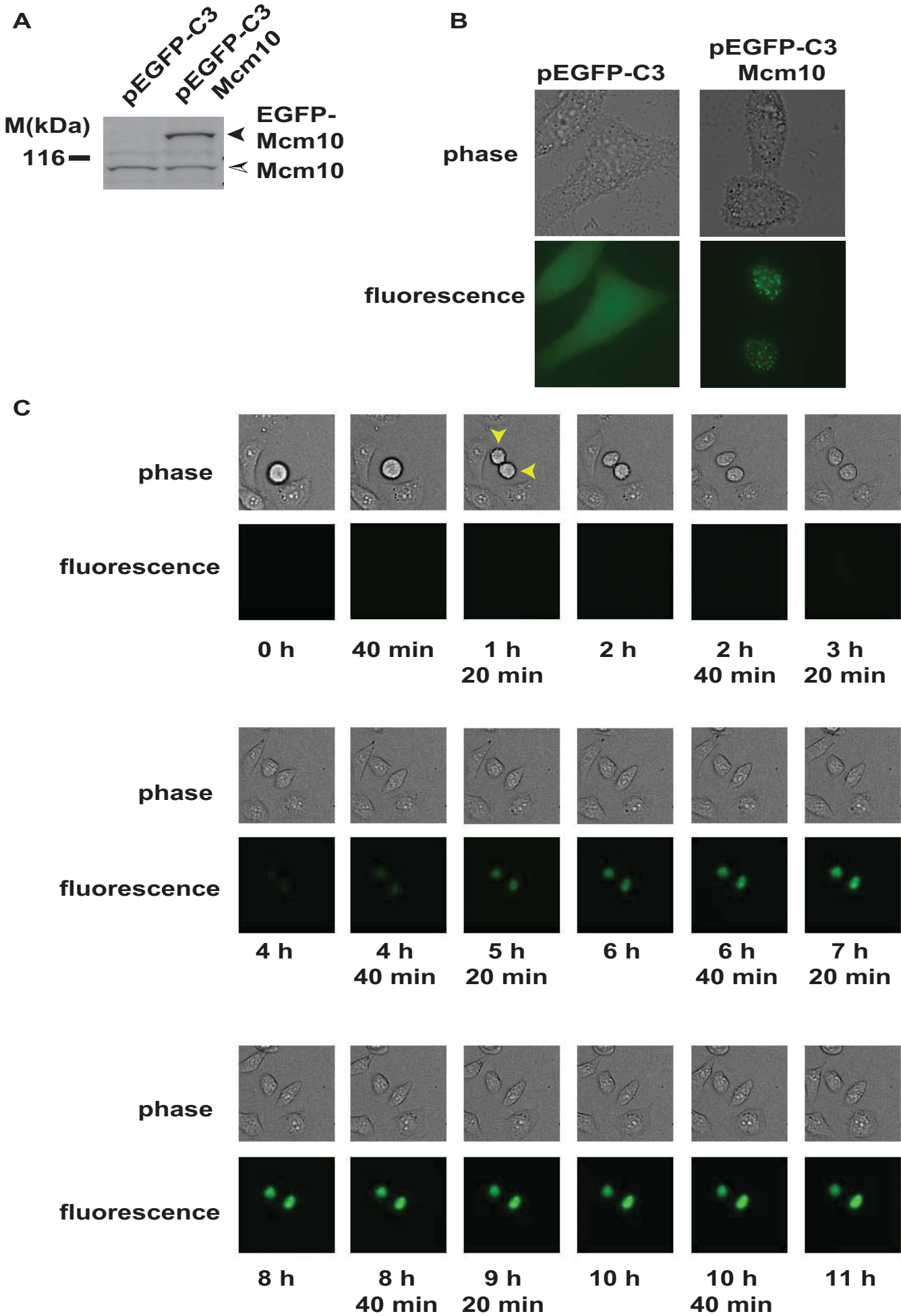

Supplement: Additional file 5 — Figure S4: Expression of GFP signal in HeLa cells transfected with either pEGFPC3 or pEGFPC3-Mcm10. (A) Transfected HeLa cells were harvested and analyzed by rabbit anti-Mcm10 antibody which identifies the mobility of the endogenous Mcm10 (shaded arrowhead) and exogenously expressed EGFP-Mcm10 (black arrowhead). (B) Two days after transfection, cells were placed on a live cell imaging stage (37°C with 5% CO2), and images were captured with a 40× objective of a Zeiss Observer Z1 inverted fluorescent microscope using an AxioCam HRm digital CCD camera. (C) Time-lapse imaging analysis of asynchronous culture of HeLa cells expressing EGFP-Mcm10. HeLa cells were transfected with pEGFPC3-ZF Mcm10 and 24 h after transfection, cells were placed on a live cell imaging stage (37°C with 5% CO2), and images were captured at 20 min intervals as described in Figure 4. Some of the representative images have been shown along with the time elapsed since the start of imaging. Top rows are phase-contrast images while the bottom rows are corresponding EGFP fluorescent images in dark field. The two arrows indicate the daughter cells after cytokinesis at 1 h 20 min. [file 1471-2121-11-84-S5.PDF]
