# Supplementary material for: Mcm10 proteolysis initiates before the onset of M-phase
Source: BMC Cell Biol. 2010 Oct 28;11:84. doi: 10.1186/1471-2121-11-84 (PMC2987893; doi:10.1186/1471-2121-11-84)
Supplement: Additional file 6 — Supplementary Methods. [file 1471-2121-11-84-S6.DOC]

**Additional file 6:**

**Supplementary Methods**

**RNAi silencing in HeLa cells**

The sequences targeting the human genes were as follows:

*APC3*, 5´ GGAAATAGCCGAGAGGTAA 3´, 5´ CAAAAGAGCCTTAGTTTAA 3´, 5´ AATGATAGCCTGGAAATTA 3´ & 5´ GCATATAGACTCTTGAAAG 3´,

*GL2*, 5´ AACGTACGCGGAATACTTCGA 3´.

*MCM10*, 5´ CTGGATTTCTACAAATAATAA 3´,

*CYCLIN A*, 5´ AAGGCAGCGCCCGTCCAACAA 3´.

Transfection of siRNA targeting endogenous genes was carried out using Lipofectamine 2000 (Invitrogen). HeLa cells were transfected with 40-80 nM siRNA duplex on three consecutive days and were harvested 24 h after last transfection to evaluate the levels of protein and mRNA by immunoblotting and reverse-transcriptase PCR respectively.

**RNA extraction and reverse-transcriptase PCR**

For RNA extraction, the cell pellet obtained from the siRNA transfected cells was resuspended in TRIzol reagent (Invitrogen, Cat.No. 15596-018) followed by chloroform addition. The RNA from aqueous layer was precipitated using isopropanol and finally resuspended in RNase free water after 70% ethanol washes. For RT-PCR, RNA was quantified using NanoDrop spectrophotometer (NanoDrop Technologies, ND-1000). cDNA synthesis was carried out using 0.25-1 µg RNA, 10 µM oligo dT_20_ primer, 1 mM dNTPs, 5X Mu-MLV reverse transcriptase buffer, RNase inhibitor (RNasin, Promega) and Mu-MLV reverse transcriptase enzyme (200 U/µl, Fermentas). The sequences of the primers used for PCR were as follows:

*APC3*, forward primer: 5´ATGACGGTGCTGCAGGAA3´,

reverse primer: 5´TTGCTGAGATCAACACAACA3´,

*BETA-2 MICROGLOBULIN*, forward primer: 5´GTTGACTTACTGAAGAATGGAGAGA3´,

reverse primer: 5´TCAATATTAAAAAGCAAGCAAGCAG3´,

**Construction of plasmids**

The primers used for cloning Mcm10 in pEGFP-C3 vector were as follows:

Forward primer:

5´GGAAGATCTCATGGATGAGGAGGAAGACAATCTG3´

Reverse primer:

5´GCCGACGTCGACTTATTTAAGGCTGTTCAGAAATTTAGC3´

The primers used for cloning ZF motif (783-843aa) of Mcm10 in pEGFP-C3 vector were as follows:

Forward primer:

5´ ATAAGAATGCGATCGCATGCAAGACGTGCGCCTATACCCAC 3´

Reverse primer:

5´ ATAGTTTAGCGGCCGCGCCCGTTCCCATTTGTAGAGGCCACA 3´

The cloning primers for generation of Mcm10 fragment in pMX-puro-NLS-HA retroviral vector were as follows:

**Mcm10 (707-770)** in pMX-puro-NLS-GFP-HA

Forward primer:

5´GGAAGATCTCCACCATGCAAGCTGAGGATGAATTGGAGCCT 3´

Reverse Primer:

5´GCCGGAATTCCATCTTTTCTTCCATTTGTTCTTT 3´

**Mcm10 (607-707)** in pMX-puro-NLS-GFP-HA

Forward primer:

5’ GGAAGATCTCCACCATGCCTCCACGGACAGGATCCGAGTTC 3’

Reverse Primer:

5’ AAGACTCAATTGTTGAGAAGAAAACATGGTGTT 3’

**Mcm10 (770-875)** in pMX-puro-NLS-GFP-HA

Forward primer:

5’ GGAAGATCTCCACCATGATGAGAAACATCAGAGAAGTG 3’

Reverse Primer:

5’ AAGACTCAATTGTTTAAGGCTGTTCAGAAATTT 3’

**Mcm10 (770-783)** in pMX-puro-NLS-GFP-HA

forward primer

5’ GATCCCCACCATGAGAAACATCAGAGAAGTGAAGTGCCGTGTCGTGACATGCG 3’

Reverse primer

5’ AATTCGCATGTCACGACACGGCACTTCACTTCTCTGATGTTTCTCATGGTGGG 3’

**Mcm10 (843-875)** in pMX-puro-NLS-GFP-HA

Forward primer:

5’ CGCGGATCCCCACCATGCGGGACGGAATGCTAAAGGAA 3’

Reverse Primer:

5’ CCGGAATTCTTTAAGGCTGTTCAGAAATTTAGC 3’

**Mcm10 (783-823)** in pMX-puro-NLS-GFP-HA

Forward primer:

5’ CGCGGATCCCCACCATGTGCAAGACGTGCGCCTATAC 3’

Reverse Primer:

5’ CCGGAATTCGATGCTTCTGTTTCCACAGG 3’

**Mcm10 (803-843)** in pMX-puro-NLS-GFP-HA

Forward primer:

5’ CGCGGATCCCCACCATGGAATACCACTGGCATGATGGT 3’

Reverse Primer:

5’ CCGGAATTCCCGTTCCCATTTGTAGAG 3’

**Mcm10 (783-803)** in pMX-puro-NLS-GFP-HA

Forward primer

5’GATCCCCACCATGTGCAAGACGTGCGCCTATACCCACTTCAAGCTGCTGGAGACCTGCGTCAGTGAGCAGCATGAAG 3’

Reverse Primer:

5’AATTCTTCATGCTGCTCACTGACGCAGGTCTCCAGCAGCTTGAAGTGGGTATAGGCGCACGTCTTGCACATGGTGGG 3’

**Mcm10 (803-823)** in pMX-puro-NLS-GFP-HA

Forward primer:

5’ CGCGGATCCCCACCATGGAATACCACTGGCATGATGGT 3’

Reverse Primer:

5’ CCGGAATTCCCGTTCCCATTTGTAGAG 3’

**Mcm10 (823-843)** in pMX-puro-NLS-GFP-HA

Forward primer

5’GATCCCCACCATGATCTCCTTGGACAGACTCCCGAACAAGCACTGCAGTAACTGTGGCCTCTACAAATGGGAACGGG 3’

Reverse Primer:

5’AATTCCCGTTCCCATTTGTAGAGGCCACAGTTACTGCAGTGCTTGTTCGGGAGTCTGTCCAAGGAGATCATGGTGGG 3’

**Mcm10 (440-607)** in pMX-puro-NLS-GFP-HA

Forward primer:

5’ GGAAGATCTCCACCATGCCAAAGAAGTTTGCCCGCAGAGGC 3’

Reverse Primer:

5’ GCCGGAATTCAGGCTGAGCAGGGGGCTGTCTTGATGA 3’

**Mcm10 (440-471)** in pMX-puro-NLS-GFP-HA

Forward primer:

5’ GGAAGATCTCCACCATGCCAAAGAAGTTTGCCCGCAGA 3’

Reverse Primer:

5’ AAGACTCAATTGAGCTGCATACGAGGCAGAAGA 3’

**Mcm10 (471-525)** in pMX-puro-NLS-GFP-HA

Forward primer:

5’ GGAAGATCTCCACCATGGCTTCAATTGCAGCAGCTGTGGCT 3’

Reverse Primer:

5’ CCGGAATTCCAGGTCCATCAGTTCCTTGAA 3’

**Mcm10 (525-607)** in pMX-puro-NLS-GFP-HA

Forward primer:

5’ GGAAGATCTCCACCATGCTGCCGACGTGTGGAGCCAGG 3’

Reverse Primer:

5’ GCCGGAATTCAGGCTGAGCAGGGGGCTGTCTTGATGA 3’

**Mcm10 (440-525)** in pMX-puro-NLS-GFP-HA

Forward primer:

5’ GGAAGATCTCCACCATGCCAAAGAAGTTTGCCCGCAGA 3’

Reverse Primer:

5’ CCGGAATTCCAGGTCCATCAGTTCCTTGAA 3’

**Mcm10 (471-607)** in pMX-puro-NLS-GFP-HA

Forward primer:

5’ GGAAGATCTCCACCATGGCTTCAATTGCAGCAGCTGTGGCT 3’

Reverse Primer:

5’GCCGGAATTCAGGCTGAGCAGGGGGCTGTCTTGATGA3’

**Mcm10 (440aa to 607aa loop out 471 to 525 aa)** in pMX-puro-NLS-GFP-HA

Forward primer:

5’GGGGTTTCTTCTGCCTCGTATGCACCGACGTGTGGAGCCAGGAACTTA 3’

Reverse Primer:

5’ TAAGTTCCTGGCTCCACACGTCGGTGCATACGAGGCAGAAGAAACCCC 3’

This retroviral plasmid containing Mcm10 fragment was then co-transfected in 293T cells along with helper plasmids that express viral VSV-G envelop and gag & pol protein, using Lipofectamine 2000 (Invitrogen). After 48 h of transfection, growth medium from the 293T cells containing viral particles was filtered through 0.45 µm filter, supplemented with 5 µg/ml polybrene and used to infect U2OS cells for 24 h. Puromycin (1 µg/ml) was then added to select the infected cells expressing Mcm10 and its different fragments. The expression of Mcm10 and its fragments was detected using anti-HA monoclonal mouse antibody.
